# From venous congestion to placental hypoxia: the underappreciated role of chronic venous disease in impaired placenta development and pregnancy health

**DOI:** 10.3389/fmed.2026.1753334

**Published:** 2026-02-05

**Authors:** Yang Zhang, Xiaotong Huang, Li Zou, Xiangwei Cheng, Xiaoxia Liu

**Affiliations:** 1 Department of Obstetrics and Gynecology, Union Hospital, Tongji Medical College, Huazhong University of Science and Technology, Wuhan, China; 2 Department of Vascular Surgery, Union Hospital, Tongji Medical College, Huazhong University of Science and Technology, Wuhan, China

**Keywords:** chronic venous disease, management, perspective, placenta, pregnancy

## Abstract

Chronic Venous Disease (CVD) is a common vascular disorder, primarily affecting the lower extremities, with a significantly higher incidence in women. Pregnant women represent a particularly high-risk population for CVD. Early screening and assessment of CVD severity and progression during pregnancy are imperative for preventing Venous Thromboembolism (VTE). Despite its high prevalence, CVD in pregnancy often remains underestimated, frequently being managed by clinicians as a localized and benign condition. However, emerging evidence suggests that CVD may exert broader systemic effects, potentially compromising placental development and fetal well-being through alterations in the maternal-placental-fetal circulation. Nevertheless, the precise correlations between CVD and a spectrum of adverse pregnancy outcomes remain unclear. Also, the standardized management strategies for CVD in pregnancies are yet to be established. This review synthesizes current literature to delineate the present understanding and identify persistent knowledge gaps in this field. Furthermore, it aims to underscore the clinical significance of CVD in pregnancy and to propose pertinent directions for future research, thereby advocating for heightened clinical awareness and more investigative efforts.

## 1 Introduction

Chronic Venous Disease (CVD) is a pathophysiological condition characterized by a constellation of symptoms and signs arising from structural or functional abnormalities in the venous system, which impairs venous return and leads to venous hypertension. CVD predominantly manifests as lower extremity venous disorders, its clinical manifestation encompasses a spectrum of features, including but not limited to heaviness, fatigue, distending pain, edema, pruritus, burning sensations, pigmentation, and muscular cramps (1). In severe cases, these can progress to varying degrees of varicose veins (VV) and even venous ulcers (1). The global prevalence of CVD in adults is estimated between 45.6 and 83.6% (2). Furthermore, CVD exhibits a marked female predilection, affecting approximately 67.5% of women. Thereinto, pregnancy is recognized as a major predisposing factor to initiate or exacerbate CVD (3). Although highly prevalent in pregnancy (affecting approximately one-third of pregnant women), the pathological implications of CVD are not confined to the lower extremities. There is growing recognition that CVD may exert systemic effects, potentially influence placental development and compromise placental function through the systemic circulation, ultimately serving as a mediator for adverse perinatal events (4–6). However, clinical awareness of the broader implications of CVD in pregnancy remains inadequate, and the correlations between CVD and multiple adverse pregnancy outcomes have yet to be fully elucidated. This review synthesizes current literature to provide a concise overview of the research landscape concerning CVD in pregnancy, bridging both basic science and clinical perspectives. Furthermore, it aims to outline future research directions, with the ultimate goal of advocating for heightened clinical vigilance and more rigorous investigation into this common yet underappreciated condition.

## 2 Impact of pregnancy on CVD

The primary etiologies and pathogenic mechanisms underlying CVD encompass: ① venous hypertension in the lower extremities, attributable to factors such as valvular incompetence, impaired venous return, and dysfunction of the calf muscle pump; ② chronic inflammatory responses; ③ compromised venous microcirculation; and ④ genetic susceptibility (1, 3). The physiological adaptations of pregnancy substantially increase CVD risk through two principal pathways (1). On one hand, the increased cardiac output and expanded total blood volume during pregnancy lead to heightened venous capacitance (7, 8). On the other hand, mechanical compression of the inferior vena cava by the gravid uterus, coupled with hormone-induced venodilation mediated by elevated levels of estrogen and progesterone, collectively impede venous return (7, 8). Consequently, pregnancy significantly promotes the initiation and progression of CVD. The prevalence of chronic venous diseases among the global adult population ranges from 45.6 to 83.6% (2). It is estimated that approximately one-third of women may develop CVD during pregnancy, with 70 to 80% of pregnant women exhibiting manifestations as early as the first trimester (9). Multiparous women face a greater risk compared to their nulliparous counterparts (9). Although a subset of patients may experience a gradual alleviation of symptoms following parturition, concomitant with the decline in estrogen and progesterone levels, the majority are left with persistent venous pathology in the lower extremities (9).

## 3 Impact of CVD on pregnancy

In pregnant women with CVD, venous valvular incompetence leads to blood reflux and results in venous hypertension and hemodynamic stasis, thereby elevating the risk of obstetric Venous Thromboembolism (VTE) (10, 11). International guidelines, including the 2020 Queensland Clinical Guideline and the 2015 Royal College of Obstetricians and Gynaecologists Green-top Guideline, consistently identify CVD as a significant risk factor for obstetric VTE, underscoring the necessity for prevention of VTE during both pregnancy and the puerperium (10, 11). Despite the common clinical perception of CVD as a localized condition, emerging evidence indicates that it constitutes a systemic state characterized by elevated levels of circulating inflammatory cytokines and oxidative stress markers, which can impair vascular and organ function (4, 5). Similarly, the pathological alterations in pregnancy complicated by CVD are not confined to the lower extremities but are disseminated systemically, potentially impairing the placental development and function via the systemic circulation (6).

Ortega et al. has dedicated considerable effort to characterizing the placental pathology in pregnancies with CVD (7). Their investigations have revealed that placentas from pregnancies with CVD exhibit increased villous density, a higher incidence of syncytial knots, and elevated levels of apoptosis (12). Further molecular analyses, including assessment of CD31, podoplanin, vascular endothelial growth factor receptor-1 (Flt-1), and placental growth factor (PlGF), demonstrated aberrant enhancement of angiogenesis and lymphangiogenesis within the placental villi, consistent with a state of chronic hypoxic stimulation (13, 14). Additionally, these placentas display upregulated expression of integrin-linked kinase (ILK) concomitant with reduced E-cadherin (15), alongside abnormal extracellular matrix remodeling evidenced by increased type III collagen content, elevated matrix metalloproteinase-9 (MMP-9) expression, and diminished elastic fibers, which may collectively disturb placental implantation and development (16, 17). Metabolomic profiling has further identified dysregulation in glycometabolism and lipid metabolism, with the placental metabolic signature mirroring that of hypoxic stress (18, 19). Collectively, these findings posit that the placentas of women with CVD undergo chronic hypoxic pathological remodeling. Notably, these pathological features bear remarkable similarities to those observed in classic placental insufficiency syndromes such as preeclampsia and fetal growth restriction, which are also characterized by placental hypoxia and impaired angiogenesis (20).

Sustained placental ischemia and hypoxia can subsequently amplify oxidative stress and inflammatory responses. Studies have confirmed that placentas from CVD-complicated pregnancies exhibit significant upregulation of NADPH oxidase 1, NADPH oxidase 2, inducible nitric oxide synthase (iNOS), and extracellular signal-regulated kinase (ERK), indicative of heightened oxidative stress (21). Concurrently, there is an increase in the expression of tetraspanins, ALG-2 interacting protein X (ALIX), and heat shock protein-70 (HSP-70) (22), dysregulation of inflammatory signaling pathways such as IGF-1/PAPP-A/STC and Wnt-1/*β*-Catenin (23), and a marked increase in NLRP3 inflammasome-mediated pyroptosis (24). Therefore, placentas in the context of CVD are characterized by a concomitant and pronounced activation of both inflammatory and oxidative stress pathway (21, 25).

Furthermore, evidence of fetal compromise is observed, with elevated levels of lipid peroxidation in the umbilical cord (26) and aberrant expression of inflammatory mediators, including allograft inflammatory factor-1 (AIF-1), interleukin-10 (IL-10), IL-12, and IL-18, alongside a decreased fetal serum pH (27, 28). These findings suggest that the placental pathology associated with maternal CVD can transmit injurious signals to the fetus via the umbilical cord and fetoplacental circulation.

In summary, CVD in pregnancy represents more than a localized disorder; it can induce chronic hypoxic placental injury, which in turn mediates a cascade of immune dysregulation and oxidative stress at the maternal-fetal interface. Most recent investigations have further identified accelerated placental cellular senescence and aberrant epigenetic regulation in these patients (29). These interconnected pathological processes collectively contribute to aberrant placental development and functional insufficiency. To provide a comprehensive overview of the molecular alterations documented in CVD-complicated placentas, we have summarized the key proteins and pathways in Table 1, which synthesizes findings from cellular senescence and epigenetic dysregulation studies (29), alongside the previously discussed hypoxic, inflammatory, and oxidative stress markers (12–19, 21–28). Given that placental malperfusion is the foundational pathology underlying various adverse pregnancy outcomes, such as preeclampsia and fetal growth restriction (20), the striking similarity between the placental pathology in CVD and that of classic placental syndromes suggests a strong clinical association. However, direct clinical evidence remains limited. Asúnsolo et al. conducted a nationwide cross-sectional study examining 2,879 pregnant women with CVD versus 8,637 matched controls, demonstrating a statistically significant association between CVD and intrapartum fetal distress (adjusted OR = 1.25, 99.5% CI = 1.05–1.50) after controlling for maternal age, BMI, gestational diabetes, and hypertension (6). While the effect size is modest, the high prevalence of CVD in pregnancy (affecting one-third of pregnant women) translates this into substantial population-attributable risk. This clinical study indicated that CVD may adversely impact fetal intrauterine development and both short- and long-term neonatal health through its deleterious effects on the placental-umbilical circuit. Nevertheless, critical limitations constrain interpretation: the cross-sectional design precludes causal inference; administrative data may suffer misclassification bias; and CVD severity, duration, and treatment were not assessed. Importantly, associations with other placental syndromes—preeclampsia, fetal growth restriction, preterm birth, and stillbirth—remain unexplored in adequately powered investigations. These findings underscore the urgent need for large-scale prospective cohort studies with rigorous CVD phenotyping (including CEAP staging), comprehensive confounder assessment, and systematic evaluation of the full spectrum of adverse pregnancy outcomes. Only through such investigations can CVD be definitively established as an independent, modifiable risk factor warranting enhanced.

**Table 1:** Molecular and histopathological alterations in placentas from CVD-complicated pregnancy.

Pathological process	Molecular/Cellular markers	Key findings	Functional implications	References
Chronic hypoxia	VEGFR-1 (Flt-1), PlGF, Podoplanin	↑ Angiogenesis and lymphangiogenesis	Adaptive response to chronic hypoxic stress	(12, 13)
	Villous density, Syncytial knots	↑ Density, ↑ Apoptosis	Morphological evidence of hypoxic injury	(11)
	Glycolysis markers, Lipid metabolism	↑ Glycolytic activity, Altered lipid profile	Metabolic adaptation to hypoxia	(17, 18)
Oxidative stress	NADPH oxidase 1/2, iNOS, ERK	↑ Expression and activity	Enhanced ROS production	(19)
	Lipid peroxidation (umbilical cord)	↑ Levels in fetal circulation	Oxidative injury transmitted to fetus	(24)
	Tetraspanins, ALIX, HSP-70	↑ Expression	Cellular stress response activation	(20)
Inflammation	IGF-1/PAPP-A/STC, Wnt-1/β-Catenin	Pathway dysregulation	Abnormal inflammatory signaling	(21)
	NLRP3 inflammasome	↑ Activation, ↑ Pyroptosis	Pro-inflammatory cell death	(22)
	AIF-1, IL-10, IL-12, IL-18 (umbilical cord)	Aberrant expression	Inflammatory signals reach fetal circulation	(25, 26)
	Fetal serum pH	↓ pH	Evidence of metabolic acidosis	(26)
ECM remodeling	Type III collagen, MMP-9	↑ Collagen III, ↑ MMP-9, ↓ Elastic fibers	Abnormal tissue architecture	(15, 16)
	ILK, E-cadherin	↑ ILK, ↓ E-cadherin	Disrupted cell adhesion and signaling	(14)
Cellular senescence	Circadian markers, HAT1, KLOTHO	Dysregulated expression, ↓ KLOTHO	Accelerated aging, Epigenetic alterations	(27)

VEGFR-1, vascular endothelial growth factor receptor-1; PlGF, placental growth factor; iNOS, inducible nitric oxide synthase; ERK, extracellular signal-regulated kinase; ALIX, ALG-2 interacting protein X; HSP-70, heat shock protein-70; IGF-1, insulin-like growth factor-1; PAPP-A, pregnancy-associated plasma protein-A; STC, stanniocalcin; MMP-9, matrix metalloproteinase-9; ECM, extracellular matrix; ILK, integrin-linked kinase; HAT1, histone acetyltransferase 1; ROS, reactive oxygen species. ↑ indicates increased/upregulated; ↓ indicates decreased/downregulated.

## 4 Clinical diagnosis and management of CVD in pregnancy

### 4.1 Clinical diagnosis of CVD in pregnancy

In the vast majority of cases, a diagnosis of CVD during pregnancy can be established through a detailed medical history and comprehensive physical examination. Traditional physical tests, such as the Trendelenburg test (for saphenous vein valve competence), the Perthes test (for deep vein patency), and the Pratt test (for perforating vein valve competence), serve primarily as initial screening tools (1). The definitive diagnostic modality for pregnancy-associated venous disorders is vascular Doppler ultrasonography. This technique is preferred due to its safety, non-invasive nature, and high accuracy in detecting venous obstruction and reflux. Ultrasonographic quantification of venous reflux time allows for the stratification of reflux severity: a reflux time between 0.5 and 1.0 s is considered diagnostic; ≥1.0 to <2.0 s indicates mild reflux; ≥2.0 to <3.0 s signifies moderate reflux; and ≥3.0 s denotes severe reflux. The assessment should also incorporate venous reflux velocity for a comprehensive evaluation (1).

### 4.2 Management strategies for CVD in pregnancy

#### 4.2.1 Lifestyle modifications

Initial management focuses on conservative measures. Patients are advised to maintain a healthy weight, wear non-restrictive clothing, and avoid prolonged periods of sitting or standing. Constipation should be managed to prevent increased intra-abdominal pressure. Regular physical activity is encouraged, and elevating the lower limbs during rest and sleep is recommended. In cases of unilateral CVD, resting in a lateral decubitus position on the unaffected side can facilitate venous return in the affected limb. Adjunctive physiotherapeutic modalities, such as foot reflexology or hydrotherapy, may also offer beneficial effects in alleviating lower extremity edema (30, 31).

#### 4.2.2 Compression therapy

Compression therapy acts by counteracting venous hypertension, enhancing the efficacy of the calf muscle pump, and improving microcirculatory hemodynamics in the skin and subcutaneous tissues. This promotes venous return, alleviates CVD symptoms, and helps prevent lower extremity VTE during pregnancy. The primary modality of compression therapy in pregnancy is graduated elastic compression stockings, which are well-tolerated and can be individually tailored based on the clinical presentation. Generally: For mild telangiectasias or minor varicose veins with minimal symptoms, stockings with a pressure of 8–21 mmHg are suitable. For symptomatic venous insufficiency or the prevention of venous ulcers, a pressure of 22–29 mmHg is recommended. For venous ulcers or severe venous insufficiency, a pressure of 30–40 mmHg is indicated. In the presence of lymphedema, stockings with a pressure >40 mmHg are advised. These recommendations are based on established clinical practice and are supported by evidence from randomized studies demonstrating the efficacy of compression therapy in pregnancy (32, 33).

A randomized study by Aleksandra et al., which recruited 51 women in their second trimester to either use compression stockings (18–21 mmHg) or serve as controls, demonstrated that stockings significantly reduced the risk of lower limb edema and venous insufficiency (*p* < 0.05) and improved the quality of life during pregnancy and postpartum (32). Corroborating these findings, Airi et al. utilized ultrasonography to measure skin thickness in 24 women at 36 weeks of gestation. They observed a significant reduction in lower limb skin thickness after two weeks of compression therapy (36 weeks: 7.47 ± 2.45 mm; 37 weeks: 7.93 ± 2.83 mm; 38 weeks: 7.15 ± 2.35 mm, *p* < 0.0001), indicating a marked alleviation of edema (33).

#### 4.2.3 Prevention of obstetric VTE

Major clinical guidelines, including the *2020 Queensland Clinical Guideline: Venous Thromboembolism (VTE) Prophylaxis in Pregnancy and the Puerperium* (34), and the *2015 Green-top Guideline from the Royal College of Obstetricians and Gynaecologists: Thromboembolic Disease in Pregnancy and the Puerperium* (35), unanimously identify varicose veins as a risk factor for VTE, mandating its inclusion in VTE risk assessment models. Early prevention, diagnosis, and treatment are paramount in reducing VTE-associated maternal mortality. Consequently, a dynamic and continuous assessment of VTE risk is required throughout pregnancy and the puerperium for women with CVD. In addition to the health promotion initiatives and compression therapy outlined above, the presence of additional VTE risk factors necessitates the timely initiation of pharmacological thromboprophylaxis with low-dose low-molecular-weight heparin (LMWH), which is the recommended pharmacological agent for VTE prophylaxis in pregnancy according to international guidelines (10, 11, 34, 35).

#### 4.2.4 Pharmacological therapy

Venotropic agents (also known as venoactive drugs) can alleviate symptoms of lower extremity edema, heaviness, and pain by enhancing venous tone, reducing capillary permeability, and promoting lymphatic and venous drainage, thereby improving the function of the calf muscle pump. Representative agents include aescin (horse chestnut seed extract), flavonoid compounds (such as diosmin), and coumarins (36–39). Although several small-scale clinical studies have not demonstrated significant teratogenic effects or clear associations with adverse perinatal outcomes for these medications, the existing evidence is insufficient to definitively guarantee the absence of adverse impacts on embryonic and fetal development. Consequently, their use is generally contraindicated during the first trimester and should be approached with caution in the second and third trimesters, following a careful risk–benefit assessment (36–38).

#### 4.2.5 Surgical intervention

Surgical treatment options for CVD, including endovenous thermal ablation, non-thermal ablation techniques (e.g., cyanoacrylate embolization, mechanochemical ablation), and traditional vein stripping, are typically not recommended during pregnancy (40). While the symptoms of CVD often ameliorate postpartum, the underlying venous dilation and architectural changes are frequently irreversible. Therefore, a comprehensive re-evaluation for potential surgical intervention should be deferred until at least two months postpartum, allowing for sufficient time for spontaneous physiological resolution (7).

## 5 Conclusion and perspectives

In summary, Chronic Venous Disease (CVD) represents a highly prevalent yet frequently underrecognized comorbidity in pregnancy. Clinical management often remains confined to empirical approaches targeting localized symptoms, overlooking the potential for this ostensibly straightforward condition to instigate impaired placental development via the maternal-placental-fetal axis, thereby compromising perinatal health and culminating in adverse pregnancy outcomes. Although the diagnosis of CVD is relatively straightforward, its chronic nature and complex clinical course significantly impact the quality of life of affected pregnant individuals. Owing to safety considerations during gestation, therapeutic interventions are primarily restricted to lifestyle modifications and compression therapy, with pharmacological and surgical options being considerably limited.

Consequently, there is an imperative for obstetricians to heighten their clinical vigilance regarding CVD. A paradigm shifts from a localized to a systemic perspective is warranted, recognizing CVD as a potential risk factor for various high-risk pregnancy syndromes. The establishment of a multidisciplinary collaborative framework, involving vascular surgery, ultrasound diagnostics, and rehabilitation medicine, is crucial. This integrated approach should focus on enhanced maternal-fetal surveillance, long-term disease management, and rigorous VTE prophylaxis to mitigate the risk of placental dysfunction syndromes, such as preeclampsia and fetal growth restriction. Furthermore, instituting structured postpartum follow-up is essential for guiding subsequent treatment strategies and ultimately improving long-term prognoses.

Despite a growing body of preclinical evidence linking CVD to placental pathologies—including ischemia-hypoxia, oxidative stress, and metabolic dysregulation—the current understanding largely remains descriptive. There is a pressing need to elucidate the detailed underlying molecular mechanisms. Moreover, clinical studies investigating the association between CVD and specific adverse pregnancy outcomes are scarce, and the evidence base guiding its management during pregnancy is of low quality. Future research must prioritize large-scale, prospective clinical studies to definitively establish the correlations between CVD and adverse pregnancy outcomes like preeclampsia, fetal growth restriction, and fetal distress. Generating robust, evidence-based data is paramount to informing and refining clinical practice guidelines.

This review synthesizes the emerging evidence linking CVD to placental pathology and highlights the need for a paradigm shift in clinical management. By bridging basic science findings with clinical observations, we aim to raise awareness of CVD as a potentially modifiable risk factor for adverse pregnancy outcomes, thereby stimulating both mechanistic research and prospective clinical investigations in this underappreciated area.
